# Development and validation of a nomogram for predicting the impact of tumor size on cancer-specific survival of locally advanced renal cell carcinoma: a SEER-based study

**DOI:** 10.18632/aging.205562

**Published:** 2024-02-19

**Authors:** Junjie Bai, Qing Lu, Yahui Wen, Tong Shangguan, Yushi Ye, Jun Lin, Rong Liu, Weizhong Cai, Jianhui Chen

**Affiliations:** 1 Department of Urology, Fujian Medical University Union Hospital, Fuzhou, China; 2 The Graduate School of Fujian Medical University, Fuzhou, China; 3 Department of Breast Surgery, Fujian Medical University Union Hospital, Fuzhou, Fujian, China; 4 Department of General Surgery, Fujian Medical University Union Hospital, Fuzhou, Fujian, China

**Keywords:** surveillance, epidemiology, and end results (SEER), locally advanced renal cell carcinoma, nomogram, prognosis, survival analysis

## Abstract

This study was aimed to integrate tumor size with other prognostic factors into a prognostic nomogram to predict cancer-specific survival (CSS) in locally advanced (≥pT3a Nany M0) renal cell carcinoma (RCC) patients. Based on the Surveillance, Epidemiology, and End Results (SEER) database, 10,800 patients diagnosed with locally advanced RCC were collected. They were randomly divided into a training cohort (*n* = 7,056) and a validation cohort (*n* = 3,024). X-tile program was used to identify the optimal cut-off value of tumor size and age. The cut-off of age at diagnosis was 65 years old and 75 years old. The cut-off of tumor size was 54 mm and 119 mm. Univariate and multivariate Cox regression analyses were performed in the training cohort to identify independent prognostic factors for construction of nomogram. Then, the nomogram was used to predict the 1-, 3- and 5-year CSS. The performance of nomogram was evaluated by using concordance index (C-index), area under the Subject operating curve (AUC) and decision curve analysis (DCA). Moreover, the nomogram and tumor node metastasis (TNM) staging system (AJCC 8th edition) were compared. 10 variables were screened to develop the nomogram. The area under the receiver operating characteristic (ROC) curve (AUC) indicated satisfactory ability of the nomogram. Compared with the AJCC 8th edition of TNM stage, DCA showed that the nomogram had improved performance. We developed and validated a nomogram for predicting the CSS of patients with locally advanced RCC, which was more precise than the AJCC 8th edition of TNM staging system.

## INTRODUCTION

There is presently an observed upward trajectory in the incidence of renal cell carcinoma (RCC), constituting approximately 90% of malignant neoplasms affecting the kidney [1]. In 2020, there were approximately 431,288 newly diagnosed cases of kidney cancer globally, and 179,368 fatalities attributed to this malignancy [2]. The most common type of RCC is clear-cell RCC (ccRCC), while other types include papillary RCC (pRCC) and chromophobe RCC and so on [3]. Of all patients diagnosed with RCC, a percentage of 30% of patients with metastatic disease present with it and a further 30% develop it after radical nephrectomy [4]. The overall stage of RCC is a significant prognostic factor.

The staging system most widely used and the main criteria for the prognosis for RCC is the 8th edition TNM system of the American Joint Committee on Cancer (AJCC) staging manual published in 2018 [4, 5]. According to the system, the classification of T1 and T2 RCC is solely based on tumor size (T1a ≤ 40 mm, 40 mm < T1b ≤ 70 mm, 70 mm < T2a ≤ 100 mm, 100 mm < T2b). Conversely, T3 and T4 are categorized according to factors including invasion of peripheral fat, fatty infiltration of renal sinus, encroachment on the pyelonephric system, attack on the renal veins and even involvement of Gerota’s fascia or the ipsilateral adrenal gland, independent of tumor size. As of now, the optimal tumor size cut-off point for staging RCC remains contentious and efforts are being made to verify the cut-off point [6, 7]. Recently, Bhindi et al. [8] made an innovative contribution by combining RCC pathology and grading and using tumor size as a direct indicator to predict RCC invasiveness. Li et al. [9] and Pecoraro et al. [10] have also confirmed that RCC invasiveness may increase with tumor size. Some studies have included locally RCC patients [11] or metastatic RCC (mRCC) patients [12], there remains a scarcity of research addressing the predictability of patients with locally advanced RCC (≥pT3a Nany M0).

In order to validate the survival outcome of malignant tumors, TNM staging is insufficient to capture their biological characteristics [13]. A variety of clinical factors may also affect RCC treatment outcomes, including sex, age, race, grade, surgical treatment, molecular characteristics, and adjuvant therapy, especially locally advanced RCC patients. From the National Cancer Institute’s Surveillance, Epidemiology, and End Results (SEER) database, we gathered clinicopathological characteristics related to prognosis in patients with locally advanced RCC. For patients with locally advanced RCC, we utilize these characteristics to create a nomogram that predicts cancer-specific survival (CSS).

## RESULTS

### Optimal thresholds for age and tumor size in RCC

**Figure 1:**
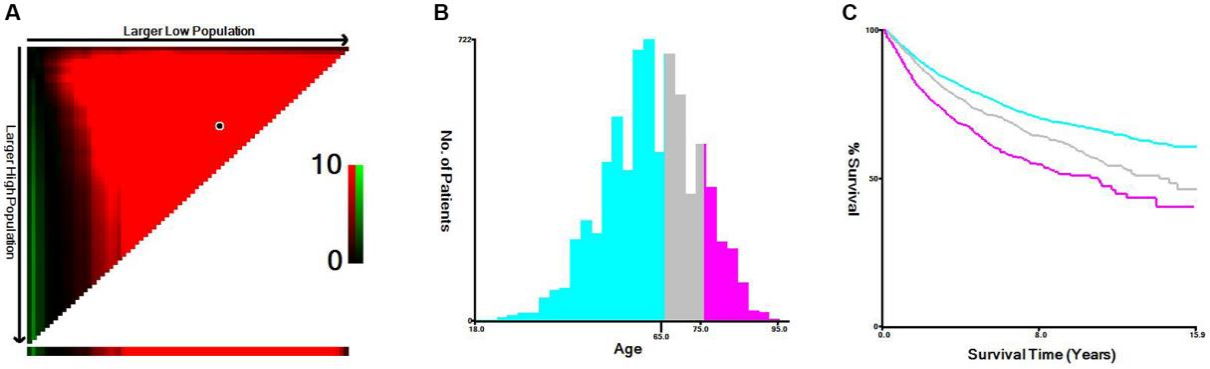
**X-tile analysis of cancer-specific survival according to age at diagnosis.** (**A**) X-tile plot of the age. (**B**) Cutoffs were depicted with histogram of the entire cohort. (**C**) Prognoses based on cutoffs are illustrated using Kaplan–Meier plots.

**Figure 2:**
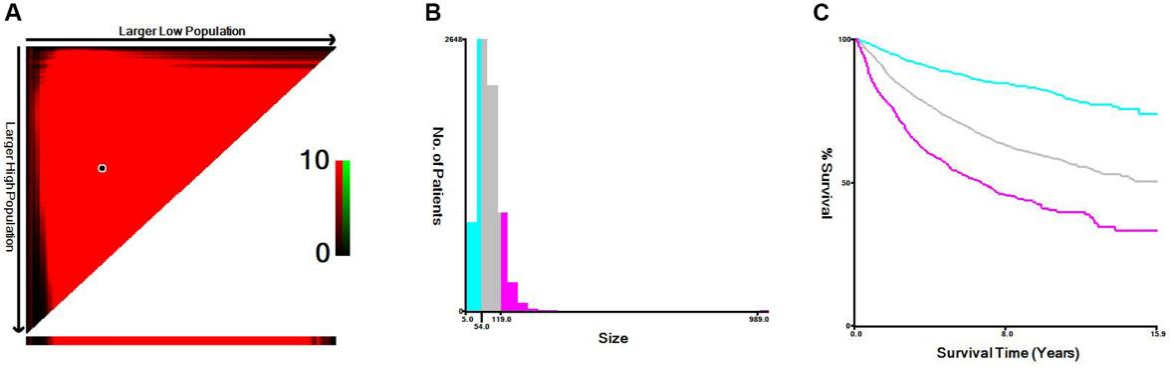
**X-tile analysis of cancer-specific survival according to tumor size.** (**A**) X-tile plot of tumor size. (**B**) Cutoffs were depicted with histogram of the entire cohort. (**C**) Prognoses based on cutoffs were illustrated using Kaplan–Meier plots.

A computer program was used to evaluate age and tumor size threshold points for patients through information about their age, tumor size, and CSS input to the X-tile software. The threshold of age was 65 years old and 75 years old, which could be divided into 3 groups: “≤65 years old”, “66–75 years old” and “>75 years old” (Figure 1). The threshold of tumor size was 54 mm and 119 mm, which could be divided into 3 groups: “<55 mm”, “55–119 mm”, and” >120 mm” (Figure 2). The data indicated that age and tumor size were strongly correlated with the prognosis of locally advanced RCC, and patients with “tumor size <54 mm” and “age ≤65 years old” had improved prognosis, and “tumor size >120 mm” and age larger than 75 years old are a negative factor that suggests a poorer prognosis.

### Baseline characteristics of the patients

**Figure 3:**
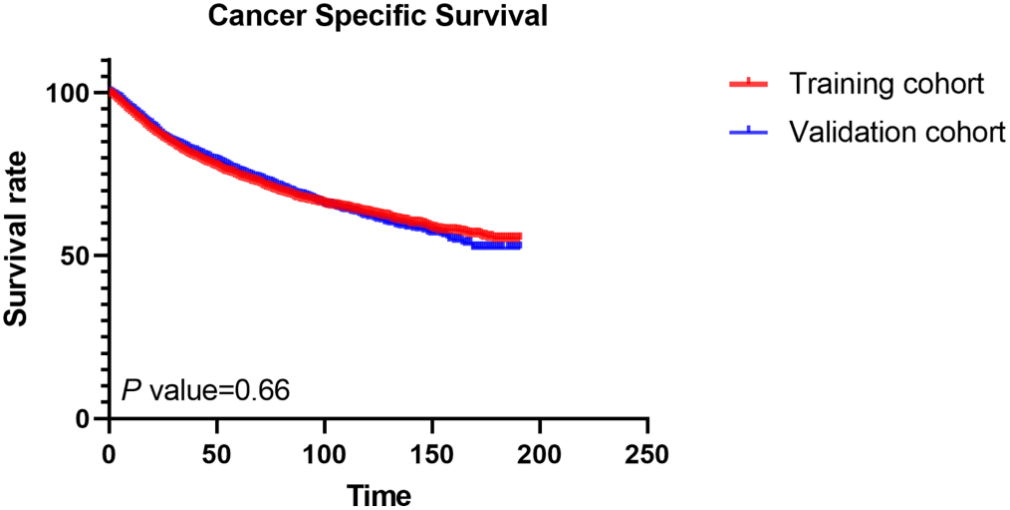
The cancer-specific survival rates in the training cohort and the validation cohort.

This study included 10,080 patients diagnosed with locally advanced RCC who met the pre-defined inclusion and exclusion criteria. A random allocation was performed, distributing 7,056 patients to the training set and 3,024 patients to the validation set. Detailed descriptive and clinical features of these patient cohorts are outlined in Table 1. Most patients were man, white, and aged ≤65 years old. CcRCC was the most common histologic type, and most tumors were between 56 and 119 mm in size. Poor differentiated tumors account for the majority of tumors and very few well-differentiated tumors. The majority of tumors were T3a and N0. Regarding treatment, most patients underwent radical nephrectomy, without surgery of lymph nodes, radiotherapy, or chemotherapy. In the training and validation sets, the 1-, 3-, and 5-year CSS rates are 93.55%, 84.64%, 79.59%, and 94.78%, 85.91%, 80.02%, respectively (*P* = 0.660, Figure 3).

**Table 1:** Characteristics between the training cohort and the validation cohort.

**Characteristics**	**Training = 7056 (%)**	**Validation = 3024 (%)**	**All patients = 10080 (%)**	***P*-value**
Age (years), *n* (%)				0.122
≤65	4133 (58.6)	1804 (59.7)	5937 (58.9)	
66–75	1899 (26.9)	828 (27.4)	2727 (27.1)	
>75	1024 (14.5)	392 (13.0)	1416 (14.0)	
Race, *n* (%)				0.450
White	6052 (85.8)	2579 (85.3)	8631 (85.6)	
Black	464 (6.6)	192 (6.3)	656 (6.5)	
Other	540 (7.7)	253 (8.4)	793 (7.9)	
Sex, *n* (%)				0.154
Male	4848 (68.7)	2121 (70.1)	6969 (69.1)	
Female	2208 (31.3)	903 (29.9)	3111 (30.9)	
Marital				0.801
Unmarried	2403 (34.1)	1022 (33.8)	3425 (34.0)	
Married	4603 (65.9)	2022 (66.2)	6655 (66.0)	
Laterality				0.257
Left	3490 (49.5)	1533 (50.7)	5023 (49.8)	
Right	3566 (50.5)	1491 (49.3)	5057 (50.2)	
Histology, *n* (%)				0.581
ccRCC	5939 (84.2)	2532 (83.7)	8471 (84.0)	
nccRCC	1117 (15.8)	492 (16.3)	1609 (16.0)	
Grade, *n* (%)				0.654
Well	310 (4.4)	137 (4.5)	447 (4.4)	
Moderate	2627 (37.2)	1161 (38.4)	3788 (37.6)	
Poor	2890 (41.0)	1220 (40.3)	4110 (40.8)	
Undifferentiated	1229 (17.4)	506 (16.7)	1735 (17.2)	
Size				0.427
<55	1980 (28.1)	827 (27.3)	2807 (27.8)	
55–119	4119 (58.4)	1759 (58.2)	5878 (58.3)	
>120	957 (13.6)	438 (14.5)	1395 (13.8)	
T-stage				0.488
T3a	5468 (77.5)	2371 (78.4)	7839 (77.8)	
T3b	1231 (17.4)	506 (16.7)	1737 (17.2)	
T3c	114 (1.6)	39 (1.3)	153 (1.5)	
T4	243 (3.4)	108 (3.6)	351 (3.5)	
N-stage				0.660
N0	6571 (93.1)	2318 (89.1)	7719 (89.1)	
N1	666 (11.0)	283 (10.9)	949 (10.9)	
N2	76 (1.1)	31 (1.0)	107 (1.1)	
Surgery, *n* (%)				0.307
None	38 (0.5)	18 (0.6)	56 (0.6)	
Ablation	10 (0.1)	6 (0.2)	16 (0.2)	
Partial nephrectomy	710 (10.1)	339 (11.2)	1049 (10.4)	
Radical nephrectomy	6298 (89.3)	2661 (88.0)	8959 (88.9)	
Surgery of LN				0.792
None	5141 (72.9)	2191 (72.5)	7332 (72.7)	
1–3	934 (13.2)	397 (13.1)	1331 (13.2)	
>4	981 (13.9)	436 (14.4)	1417 (14.1)	
Chemotherapy				0.385
No	6541 (92.7)	2818 (93.2)	9359 (92.8)	
Yes	515 (7.3)	206 (6.8)	721 (7.2)	
Radiotherapy				0.268
No	6966 (98.7)	2977 (98.4)	9943 (98.6)	
Yes	90 (1.3)	47 (1.6)	137 (1.4)	

Abbreviations: ccRCC: clear-cell renal cell carcinoma; nccRCC: non-clear-cell renal cell carcinoma; LN: lymph node.

### Prognosis and survival outcomes of locally advanced RCC patients with different tumor sizes

To further demonstrate clinical significance of this threshold for tumor size, we conducted a comparison of the variations in tumor characteristics across the three categories. According to our analysis, a larger tumor size group is linked to a younger age, a more severe tumor grade, a higher prevalence of being unmarried, positive local lymph nodes, an advanced T-stage, and a higher proportion of patients who underwent radical nephrectomy, lymph node dissection, chemotherapy, and radiotherapy (Table 2, all *P* < 0.05).

**Table 2:** Demographic and tumor characteristics at baseline stratified by tumor size.

**Characteristics**	**<55 mm (%)**	**56-119 mm (%)**	**>120 mm (%)**	**Log-rank**	***P*-value**
Total	2807	2878	1395		
Age (years), *n* (%)				153.489	<0.001^*^
≤65	1519 (54.1)	3461 (58.9)	957 (68.6)		
66–75	835 (29.7)	1576 (26.8)	316 (22.7)		
>75	453 (16.1)	841 (14.3)	122 (8.7)		
Race, *n* (%)				14.566	0.001^*^
White	2414 (86.0)	5057 (86.0)	1160 (83.2)		
Black	179 (6.4)	356 (6.1)	121 (8.7)		
Other	214 (7.6)	465 (7.9)	114 (8.2)		
Sex, *n* (%)				0.023	0.881
Male	1876 (66.8)	4115 (70.0)	978 (70.1)		
Female	931 (33.2)	1763 (30.0)	417 (29.9)		
Marital				6.901	0.009^*^
Unmarried	920 (32.8)	2010 (34.2)	495 (35.5)		
Married	1887 (67.2)	3868 (65.8)	900 (64.5)		
Laterality				0.134	0.714
Left	1397 (49.8)	2902 (49.4)	724 (51.9)		
Right	1410 (50.2)	2976 (50.6)	671 (48.1)		
Histology, *n* (%)				43.992	<0.001^*^
ccRCC	2231 (79.5)	5171 (88.0)	1069 (76.6)		
nccRCC	576 (20.5)	707 (12.0)	326 (23.4)		
Grade, *n* (%)				401.927	<0.001^*^
Well	215 (7.7)	208 (3.5)	24 (1.7)		
Moderate	1417 (50.5)	2069 (35.2)	302 (21.6)		
Poor	985 (35.1)	2484 (42.3)	641 (45.9)		
Undifferentiated	190 (6.8)	1117 (19.0)	428 (30.7)		
T-stage				264.035	<0.001^*^
T3a	2494 (88.8)	4467 (76.0)	878 (62.9)		
T3b	265 (9.4)	1152 (19.6)	320 (22.9)		
T3c	15 (0.5)	91 (1.5)	47 (3.4)		
T4	33 (1.2)	168 (2.9)	150 (10.8)		
N-stage				516.096	<0.001^*^
N0	2730 (97.3)	5485 (93.3)	1187 (85.1)		
N1	69 (2.5)	331 (5.6)	171 (12.3)		
N2	8 (0.3)	62 (1.1)	37 (2.7)		
Surgery, *n* (%)				NA	NA
None	13 (0.5)	29 (0.5)	14 (1.0)		
Ablation	14 (0.5)	2 (0.1)	0 (0.0)		
Partial nephrectomy	790 (28.1)	243 (4.1)	16 (1.1)		
Radical nephrectomy	1990 (70.9)	5604 (95.3)	1365 (97.8)		
Surgery of LN				54.817	<0.001^*^
None	2509 (89.4)	4148 (70.6)	675 (48.4)		
1–3	161 (5.7)	857 (14.6)	313 (22.4)		
>4	137 (4.9)	873 (14.9)	407 (29.2)		
Chemotherapy				141.872	<0.001^*^
No	2705 (96.4)	5461 (92.9)	1193 (85.5)		
Yes	102 (3.6)	417 (7.1)	202 (14.5)		
Radiotherapy				95.508	<0.001^*^
No	2792 (99.5)	5795 (98.6)	1356 (97.2)		
Yes	15 (0.5)	83 (1.4)	39 (2.8)		

Abbreviations: ccRCC: clear-cell renal cell carcinoma; nccRCC: non-clear-cell renal cell carcinoma; LN: lymph node; ^*^P < 0.05.

Univariate Cox analysis conducted in the training cohort disclosed that in addition to tumor size, age, race, marital status, histology, grade, T-stage, N-stage, lymph node dissection, surgery, radiotherapy and chemotherapy were also determined to be statistically relevant prognostic factors (Table 3, *P* < 0.05). Multivariate Cox analysis identified prognostic factors for patient survival by examining all relevant factors. Through the implementation of multivariate cox analysis on the training cohort, it was determined that race, age, marital status, tumor size, histology, tumor grade, T-stage, N-stage, surgery, chemotherapy and radiotherapy were identified as autonomous risk factors in relation to the CSS of individuals with locally advanced RCC (as shown in Table 3, *P*-values < 0.05).

**Table 3:** Univariate and multivariable analysis of factors associated with cancer-specific survival in locally advanced RCC patients.

**Variables**	**Univariate analysis**	***P*-value**	**Multivariate analysis**	***P*-value**
	**HR (95% CI)**		**HR (95% CI)**	
Age (years)				
≤65	Reference		Reference	
66–75	1.300 (1.168–1.446)	<0.001^*^	1.443 (1.296–1.607)	<0.001^*^
>75	1.826 (1.615–2.063)	<0.001^*^	2.189 (1.929–2.483)	<0.001^*^
Race, *n* (%)				
White	Reference		Reference	
Black	1.301 (1.095–1.546)	0.003^*^	1.299 (1.090–1.549)	0.004^*^
Other	0.831 (0.690–1.000)	0.051	0.777 (0.645–0.937)	0.008^*^
Sex, *n* (%)				
Male	Reference			
Female	0.980 (0.888–1.082)	0.695		
Marital				
Unmarried	Reference		Reference	
Married	0.862 (0.784–0.949)	0.002^*^	0.884 (0.802–0.974)	0.013^*^
Laterality				
Left	Reference			
Right	1.025 (0.936–1.123)	0.591		
Histology, *n* (%)				
ccRCC	Reference		Reference	
nccRCC	1.410 (1.259–1.579)	<0.001^*^	1.152 (1.022–1.299)	0.021^*^
Grade, *n* (%)				
Well	Reference		Reference	
Moderate	1.308 (0.950–1.801)	0.100	1.153 (0.836–1.589)	0.385
Poor	2.467 (1.802–.3.77)	<0.001^*^	1.808 (1.318–2.480)	<0.001^*^
Undifferentiated	5.555 (4.043–7.633)	<0.001^*^	3.245 (2.350–4.480)	<0.001^*^
Size				
<55	Reference		Reference	
55–119	2.569 (2.241–2.944)	<0.001^*^	1.824 (1.580–2.106)	<0.001
≥120	4.617 (3.950–5.396)	<0.001^*^	2.474 (2.090–2.927)	<0.001
T-stage				
T3a	Reference		Reference	
T3b	1.800 (1.623–1.996)	<0.001^*^	1.533 (1.379–1.704)	<0.001
T3c	2.709 (2.017–3.639)	<0.001^*^	2.176 (1.616–2.930)	<0.001
T4	4.832 (4.065–5.742)	<0.001^*^	2.477 (2.056–2.985)	<0.001
N-stage				
N0	Reference		Reference	
N1	4.331 (3.777–4.967)	<0.001^*^	2.553 (2.207–2.953)	<0.001^*^
N2	7.091 (5.558–9.047)	<0.001^*^	3.157 (2.427–4.106)	<0.001^*^
Surgery, *n* (%)				
None	Reference		Reference	
Ablation	0.100 (0.24–0.420)	0.002^*^	0.875 (0.204–3.757)	0.857
Partial nephrectomy	0.038 (0.024–0.420)	<0.001^*^	0.305 (0.184–0.506)	<0.001^*^
Radical nephrectomy	0.136 (0.093–0.198)	<.001^*^	0.536 (0.349–0.824)	0.004^*^
Surgery of LN				
None	Reference		Reference	
1–3	1.818 (1.612-2.051)	<0.001^*^		0.102
>4	1.906 (1.694–2.145)	<0.001^*^		0.158
Chemotherapy				
No	Reference		Reference	
Yes	4.143 (3.227–5.318)	<0.001^*^	1.545 (1.336–1.785)	<0.001^*^
Radiotherapy				
No	Reference		Reference	
Yes	2.647 (2.316–3.024)	<0.001^*^	2.358 (1.829–3.039)	<0.001^*^

Abbreviations: ccRCC: clear-cell renal cell carcinoma; nccRCC: non-clear-cell renal cell carcinoma; LN: lymph node; ^*^P < 0.05.

### Development and validation of nomogram

**Figure 4:**
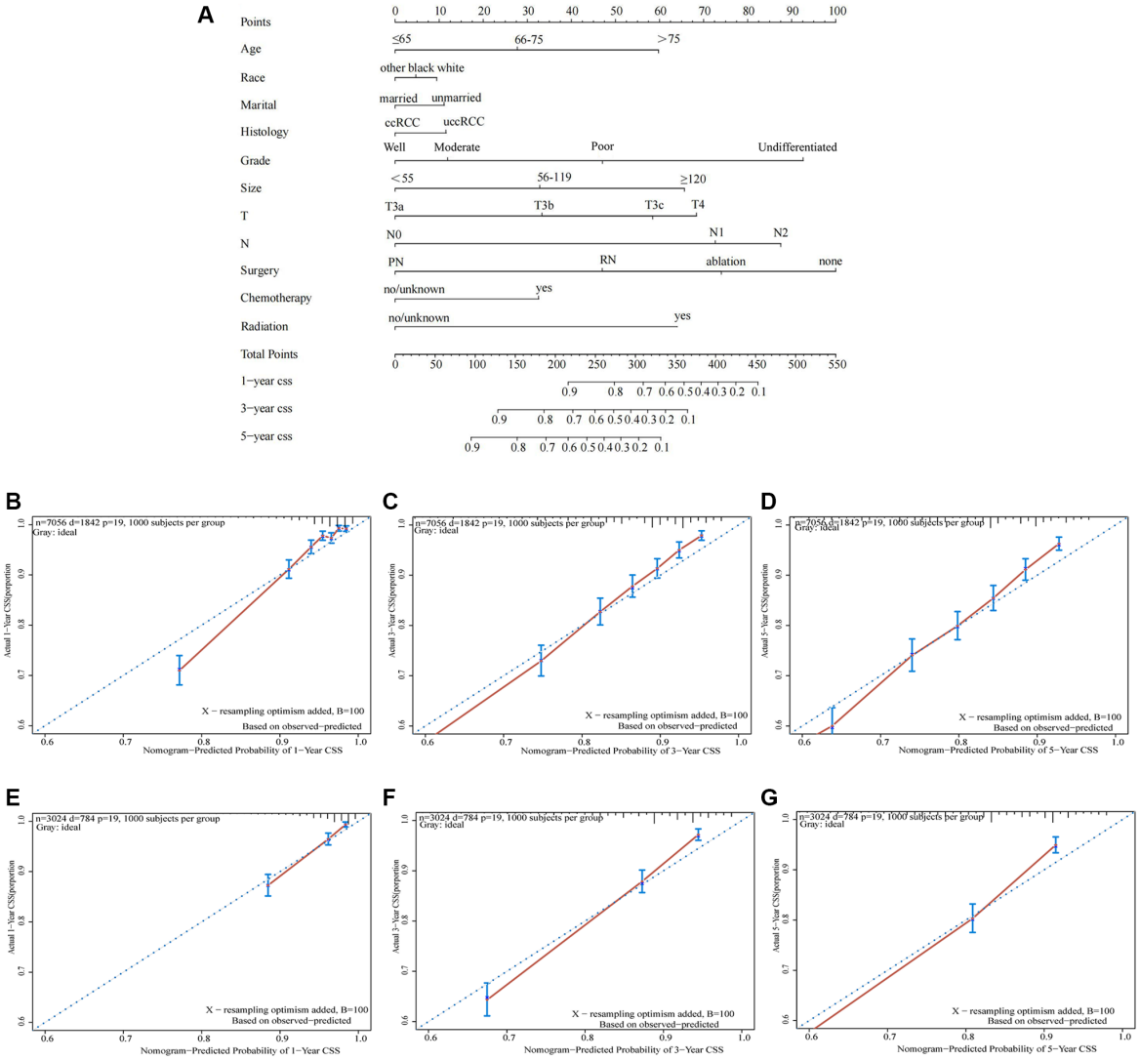
**A nomogram predicting 1-, 3- and 5-year CSS of patients with locally advanced RCC.** (**A**) Calibration plots of the relationship between predicted probabilities and actual values based on nomograms. (**B**–**D**) calibration curves for 1-year, 3-year and 5-year CSS in the training cohort; (**E**–**G**) calibration curves for 1-year, 3-year and 5-year CSS in the validation cohort).

**Figure 5:**
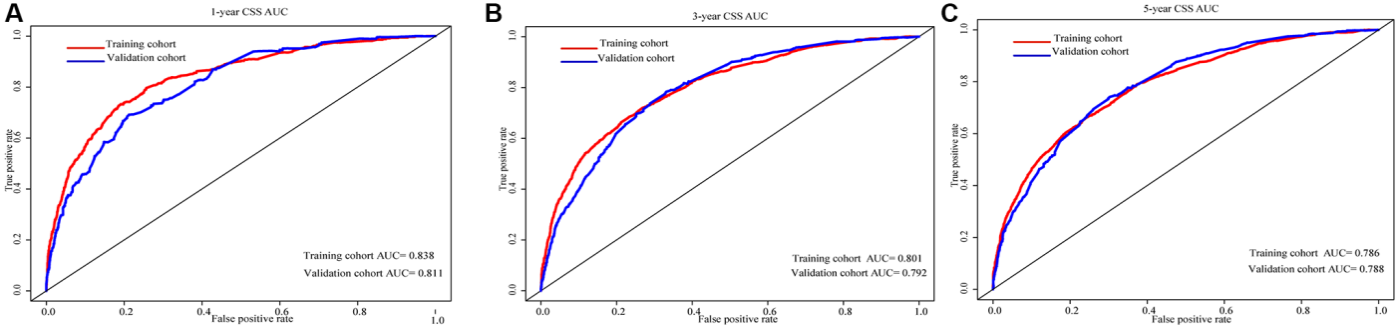
AUC for predicting 1-, 3-, and 5-year CSS in the training cohort and the validation cohort (**A**–**C**).

**Figure 6:**
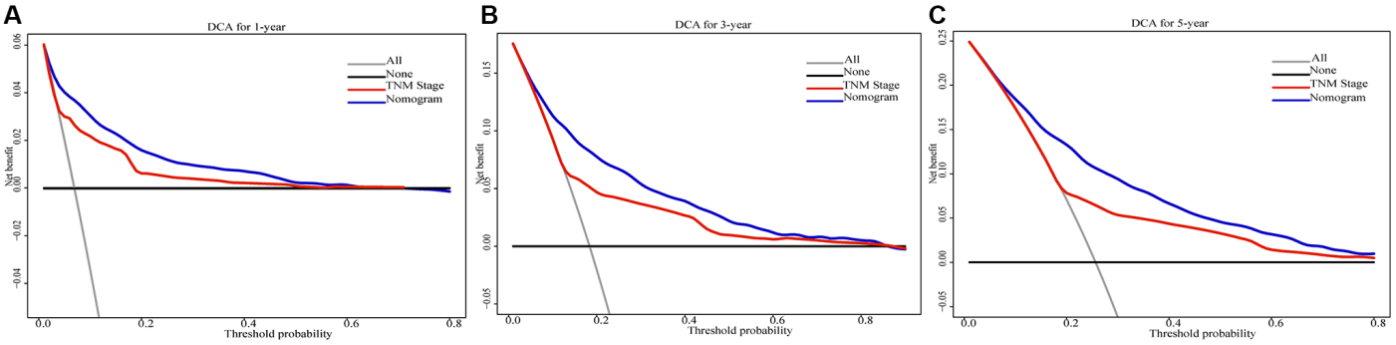
Decision curve analysis of the 1-, 3-, and 5-year CSS nomogram compared with that of AJCC 8th edition TNM stage in the validation cohort (**A**–**C**).

In training cohort, prognostic nomograms were developed for locally advanced RCC using the eleven prognostic variables which are independent for CSS (Figure 4A). In both training and validation cohorts, a robust agreement between predicted and observed survival was evident in the calibration curve of the nomogram. (Figure 4B–4G). The nomogram conveyed the impact of each variable on survival through the length of its corresponding line. According to the nomogram, the main factor influencing CSS was surgery, with tumor grade and N stage being the next significant contributors. On the other hand, race, marital status, and histology had the least impact on survival outcomes. Importantly, tumor size's impact on prognosis was essentially equivalent to that of T stage and age, both of which were widely acknowledged as crucial determinants in the prognosis of locally advanced RCC. By utilizing the survival probability scales in the nomogram, we successfully computed the cumulative risk score to estimate the CSS for 1, 3, and 5 years. Both internal and external evaluations were conducted, assessing using C-index and ROC curves (Figure 5). In training cohort, the CSS nomogram had a C-index of 0.753 (95%CI: 0.741–0.764), while in validation cohort, it was 0.751 (95%CI: 0.734–0.768). In training cohort, the nomogram yielded AUC values of 0.838, 0.801, and 0.786 for predicting 1-, 3-, and 5-year CSS rates. In validation cohort, AUC values were 0.811, 0.792, and 0.788. Next, to further contrast the nomograms with the AJCC TNM stage, DCA was performed in CSS. Within DCA, the nomograms CSS demonstrated greater efficacy compared to the TNM. In the meantime, the CSS nomogram also displayed greater statistical power. The CSS nomogram also demonstrated greater statistical power in relation to the TNM stage (AJCC 8th edition) (Figure 6).

## DISCUSSION

While the general outlook for kidney cancers is favorable [14–16], certain forms of RCC still exhibit an unfavorable [17, 18]. Primary tumor size is an important clinical feature [19–21], and one of the most important factors in TNM staging [22]. The protective factor of small tumor (<4 cm) as the overall survival time of T1-T4 mRCC patients was verified in the study cohort of Jiang and DiNatale and Jiang found that 1 cm increase in tumor size was associated with a 3.8% increased probability of death. (*P* < 0.001) [12, 23]. However, the above studies are based on TNM staging of tumor size analysis. We stratified tumor size (“<55 mm”, “55–119 mm”, and “>120 mm”) according to CSS in patients with stage T3-T4, and found that patients with smaller tumor size (<55 mm) in the cohort had longer CSS. Furthermore, researches have indicated that the age factor significantly influences the likelihood of survival for different types of cancers [24, 25]. We stratified locally advanced RCC patients with age and found that the younger the cohort (≤65 years old), the longer the CSS (mean, 140 months, vs. 127 months, vs. 111 months, *P* < 0.001). When performing a multivariate analysis, large tumor (>120 mm) and older age (>75 years old) were confirmed to be risk factors. These findings align with the earlier outcomes reported by Nelson et al. [26]. In contrast, a meta-analysis examining the predictability of benign kidney tumor revealed that age did not emerge as a standalone predictor [27]. The present study revealed that patients with small tumor size (<55 mm) had lower tumor grades, T stages, and N stages. Similar to the heterogeneity of survival outcomes highlighted by previous studies, in addition to being related to the infiltrative character of the renal tumor [28], we considered that it may also be related to tumor size and age [12, 29]. Hence, optimal cut-off values for tumor size and age were established as prognostic indicators in locally advanced RCC patients.

The TNM staging system remains a globally accepted guideline for forecasting the prognosis of RCC. Nevertheless, the TNM system primarily relies on size, the existence of LN metastasis, and distant metastasis. Most previous studies were also using the TNM model, which did not include all relevant factors, especially tumor-related factors. In recent years, the nomogram has become more popular in predicting tumor patients with higher accuracy than the conventional staging system [13]. The nomogram includes various characteristic factors like age, ethnicity, marital status, and tumor grade in a quantitative model. This model enables the estimation of survival rates by considering the unique attributes of patients [30, 31]. In previous study, the prognostic impact of RCC with T3N0M0 is relied on the AJCC TNM system [32]. Hence, our objective was to merge these tumor-associated variables in order to enhance the prognostic accuracy after surgery for individuals with locally advanced RCC. To overcome this issue, we firstly used the X-Tile procedure to identify the optimal cut-off values of age and tumor size, and develop a more precise and valid survival model based on tumor size and age. According to our findings, patients aged >75 years, white race, single, non-clear cell carcinoma, high pathological grade, tumor ≥120 mm, high T-stage, high N-stage, not treated with surgery, receiving radiotherapy and chemotherapy had a poorer prognosis. Among these options, partial nephrectomy (PN) had the most favorable outcome, followed by radical nephrectomy (RN) with a relatively good prognosis, local tumor resection had a poor prognosis, and failure to undergo surgery had the worst prognosis. It aligns with the majority of research findings. Earlier studies have discovered that individuals categorized as widowed, separated/divorced, and never-married have a greater likelihood of not receiving surgical treatment. However, an increased risk of higher T-stage and tumor grade does not result from this. [33]. Married is a positive prognostic factor for patients with both localized and mRCC [34]. In patients with locally advanced RCC, our research indicates that being single is associated with a reduced (CSS). Gu et al. categorized age into young (≤67 years), middle-aged (68 ~ 80 years), and old (>80 years) groups and demonstrated the independent prognostic factor of age in mRCC [35]. In comparison, the subgroups in our study were younger, which may be related to the staging of the disease. In addition, age was also an important factor influencing marital status, with more unmarried people at older ages, which may be related to widowhood. Wang et al. crafted a nomogram for predicting CSS in ccRCC patients. The nomogram demonstrated commendable efficacy in forecasting cancer-specific survival across various stages, spanning from T1 stage to T4 stage, and encompassing diverse metastatic sites [36]. However, a notable limitation which was confined to discrete categories of 70 mm and 100 mm. Furthermore, the study did not delve into a more nuanced staging system. In our investigative endeavor, we sought to address this limitation by implementing a refined staging system, categorizing into T3a, T3b, and T3c.

This is the initial investigation to create nomograms for locally advanced RCC that are stratified by tumor size. We validated the accuracy and predictive power of our nomograms using AUC, C-index, and DCA to forecast CSS. Additionally, our nomograms exhibit superior predictive accuracy in contrast to both the AJCC TNM staging system and the morpho grams created for locally advanced RCC. The potential worth of these maps extends to both academic research and clinical practice, as they show a satisfactory ability to differentiate patients. However, it is crucial to acknowledge that although our study provides a pragmatic method for assessing tumor risk, it might not have a substantial impact on current management of RCC and may only be useful in particular circumstances. Overall, our findings are crucial for assessing the outlook of individuals with regionally advanced RCC.

However, there are some limitations in this study. First, some crucial factors like smoking and alcohol intake were absent from the SEER database. The prognosis may be greatly influenced by these variables. Nevertheless, we attempted to include a number of key factors, such as the type of tumor, and other basic patient information in the database to reduce the potential impact of these variables. Furthermore, due to the nature of this study being retrospective, it is not possible to entirely eradicate selection bias. Future prospective studies are warranted to validate our findings. Finally, in order to determine whether our model is accurate and generalizable, we need to conduct external validation. In addition, we classify pRCC, unclassified RCC (URCC) and chromophobe RCC as nccRCC, which may lead to compromised prognostic outcomes for patients.

## CONCLUSION

The study investigated the predictive factors of locally advanced RCC patients. The age, size, grade, TNM stage, treatment was identified as individual risk factors that impact patients' CSS. A nomogram was built with high precision and reliability to forecast the CSS of locally advanced RCC patients, aiding clinicians and patients in making informed clinical choices.

## METHODS

### Patients and selection criteria

Data extraction from the SEER database for locally advanced RCC patients involved the use of SEER*Stat software (version 8.4.0.1). The extracted data included demographic variables, tumor characteristics such and further treatment. No need for informed consent or ethics committee approval as the SEER database is accessible to the public. The research was carried out following the guidelines of the Helsinki Declaration (revised in 2013).

**Figure 7:**
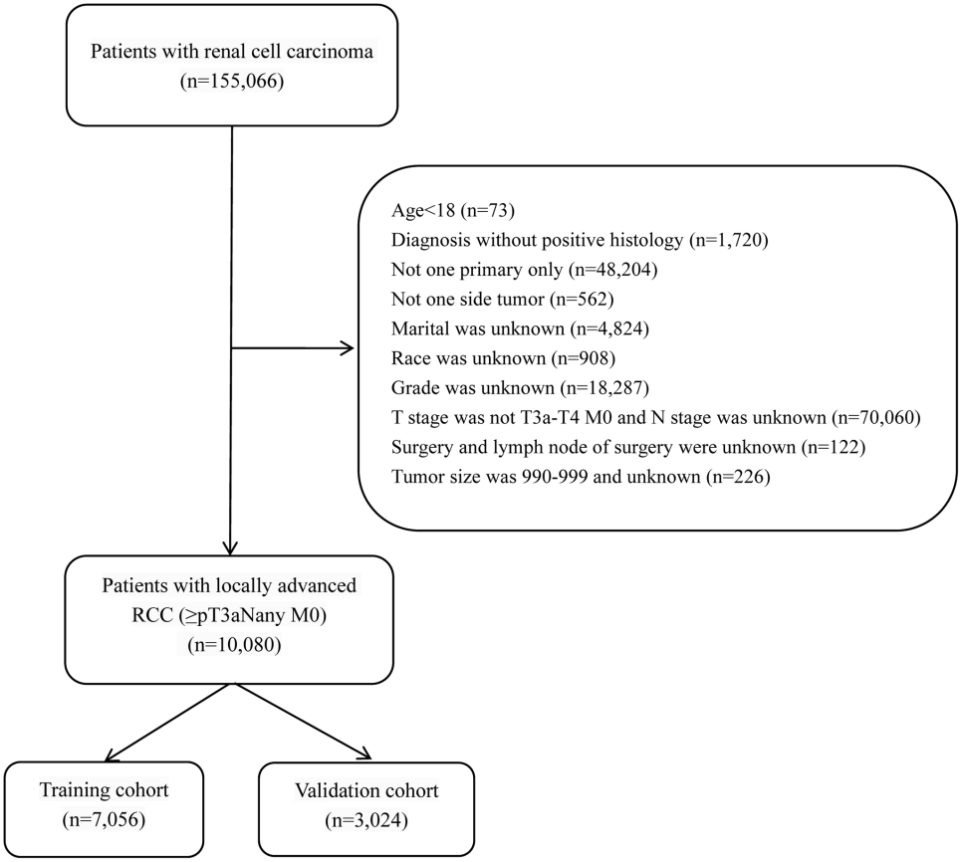
Flowchart displaying the extraction process of locally advanced RCC (≥pT3a Nany M0) cases in SEER database.

The inclusion criteria for this study were as follows: (1) patients who were diagnosed with locally advanced RCC (≥pT3a Nany M0). (2) age ≥18 years. (3) unilateral primary RCC. (4) individuals who had a complete record of cancer-specific survival months. (5) positive histologically confirmed diagnosis with histology codes 8260/3, 8310/3, 8316/3, 8317 /3, 8318/3, 8319/3, 8323/3, 8510/3 and (6) individuals with only one primary tumor. Exclusion criteria included the following: (1) patients who had unknown information regarding their age, race, marriage, tumor size, histological type, grade, surgery, T-stage, N-stage, M- stage, radiotherapy, etc. (2) individuals lacking full follow-up. Since the histological type was mostly ccRCC, the subhistological subtypes were divided into ccRCC and nccRCC. The flowchart of localized advanced RCC (≥pT3a Nany M0) case extraction from the SEER database was presented in Figure 7.

### Clinical variables extracted for analysis

To establish and validate the nomogram, the SEER database extracted all eligible locally advanced RCC patients, who were randomly divided into training (70%) and validation (30%) cohorts. The main primary endpoint was CSS, and the duration of CSS was calculated from the time of diagnosis to the occurrence of death caused by locally advanced RCC. We utilized X-tile software (Version 3.6.1, https://medicine.yale.edu/lab/rimm/research/software/) in conjunction with patients' CSS to determine the most suitable thresholds for size and age. These thresholds, along with other variables, were included in the statistical analysis. In terms of statistics, the chi-square test was employed to compare all the categories in the training and validation groups. In the training set, the tumor size was stratified, and Kaplan-Meier curves were employed to identify factors associated with CSS of locally advanced RCC. The log-rank test was utilized to analyze discrepancies between the curves.

### Construction and validation of the nomogram

To examine predictive factors linked to locally advanced RCC and estimate hazard ratios (HR) and 95% confidence intervals (CI), we employed both univariate and multivariate Cox models. After analyzing the results, we utilized the R software (version 3.6.1) to create the nomogram using various packages. The newly developed nomogram was evaluated using the validation group. The concordance index (C-index) was used to evaluate the agreement between the nomogram prediction and observed outcomes. The calibration plot visually compared the predicted prognosis by the nomogram with the actual outcomes. The evaluation of sensitivity and specificity was done using the receiver operating characteristics curve (ROC), specifically by calculating the area under the curve (AUC). Moreover, the nomogram model's efficacy was also evaluated in decision curve analysis (DCA) when compared to the TNM stage. Statistical analysis was conducted using R software version 3.6.1 and SPSS 26.0. *P*-value of less than 0.05 was deemed statistically significant.

### Data availability statement

Publicly available datasets were analyzed in this study. This data can be found here: https://seer.Cancer.gov/.
